# Mr. Armstrong, on Hydrophobia

**Published:** 1808-12

**Authors:** Charles Armstrong

**Affiliations:** Upper Charlotte Street, Fitzroy Square


*Though the two following Papers are controversial, yet we are sure our Readers would not excuse the omission of so much useful information as they contain. The authors too may claim the privilege of defending themselves; but we must again urge the necessity of declining to receive any remarks, whether anonymous or otherwise, if mixed with severity or petulance.*

### *To the Editors of the Medical and Physical Journal.*

Gentlemen,

An anonymous correspondent in your last number, who has done me the honour to animadvert on my paper, on the prevention of hydrophobia, has prefaced his observations with a little flourishing about hasty assumption, new remedies, and medical absurdity, which may be very amusing, but, unfortunately, is wholly inapplicable to the subject in question. It is, in truth, a sort of random firing from behind a skreen, without aim or object.

A plain recital of a series of facts leading to a certain conclusion, can be no hasty assumption; nor can a practice as old as the time of Celsus, be fairly classed with new remedies; medies; and, as to medical absurdity, C. B. himself, by his eulogy on my practice, pronounces honourable acquittal. My sole motive in requesting your insertion of that paper, was, to counteract the effect of the two unfortunate cases alluded to, which I considered as tending to prove the inutility of the destruction of the part bitten by a rabid animal, and, consequently, to check the only practice that has been fairly established as a prophylactic in one of the most dreadful of human calamities.

I have little time and less inclination for controversy, but as the subject is of some moment, I will, with your permission, offer a few observations on the doubts and objections of this anonymous author.

In my narrative I have said, "The dog was following a proud bitch, and on the boy coming in his way, he seized him by the heel." The pursuit the dog was engaged in, was mentioned, not altogether with the intent of accounting for his biting the boy at that particular time, but rather to prove the progress of the disease in the animal, by the subsequent act of biting and tearing the same bitch two days after. C. B. however, seems disposed to think the interruption to the amour, an offence of a grave nature, fully adequate to call forth the resentment of the dog; and this might be very true, had the boy interfered on the occasion; but the fact is, he was accidentally crossing the yard, and offered no interruption to the animal whatever.

But C. B. doubts the existence of disease in the dog, at the time he bit Master Hill, because there is no proof of his being hydrophobous at that period. By the term *hydrophobous,* I presume the gentleman would have us understand *rabid,* because the single symptom hydrophobia, as you have justly observed in a note, is not essentially necessary to constitute that degree of rabies in the animal, which is capable of communicating hydrophobia to the human species. Further, if C. B. will have the goodness carefully to examine the cases of genuine hydrophobia, succeeding the bite of rabid animals, published within the last fifty years, he will find that a considerable number of the unhappy persons were bitten by dogs not supposed at the time to be mad.

The next objection started by C. B proves, I am sorry to say, his complete want of information on the subject. It is conveyed in these words. "I doubt whether any dog ever lived four days after having begun to be affected with symptoms of the true rabies canina." This has certainly

 happened happened within my own experience, but I will take the proof from the most incontestible source.

Mr. Reynel, whose observations appeared in several periodical works in the course of the last year, and whose extensive knowledge in whatever relates to the economy of sporting dogs, is universally admitted, says, " I have seen dogs eat, and lap water, the day before their death, which generally happens between seven and ten days after the first symptom has appeared." He also observes, that "mad dogs appear to be capable of communicating the infection early in the disorder, as soon as they begin to quarrel with or bite other dogs." Here then, gentlemen, we have contemporary authority of no ordinary estimation, to prove, that hydrophobia is no necessary symptom of rabies in dogs, and also perfectly to remove all uncertainty, respecting the number of days a dog usually survives the first symptom of rabies. Hence, C. B. may learn, that instead of dying within *four* days, dogs generally live from *seven* to *ten* days, after being affected with symptoms of the true rabies canina.

Another doubt arises from the early period at which, after having been bitten by the dog, the cat and the bitch became affected. Let us see how that stands. Mr. Reynel has never known a mad dog shew symptoms of the disease in less time after the bite than ten days. It was the tenth day that the cat became affected, and bit the girl, and the thirteenth day when the bitch first refused food. It was in the hottest season, and the air at a high degree of temperature, capable of giving activity to the virus. From all the foregoing circumstances, I feel confident of having plain truth on my side, when I assert, that in these instances, there is no deviation from the common course of infection.

Having briefly, and I hope satisfactorily, replied to the doubts and objections of your unknown correspondent, I would ask, if he is serious in comparing the ridiculous amulet he calls a camphor bag, with the excision or destruction of the part bitten by a rabid animal? To my limited capacity, the incongruous association savors more of absurdity than science. I must likewise confess my want of discernment, to discover the *valuable information* contained in the tale of the drunken footman, from Dr. James. If this sort of reasoning carries any point whatever, its effect must be to damp or restrain every exertion to relieve, by representing all endeavours as futile or nugatory.

In the science of medicine, a moderate share of scepticism cism is laudable and proper; but what are we to think of a man, who positively rejects facts, and wilfully refuses conviction? Such, if he actually perused the whole of the case he attempts to criticise, is your Correspondent C. B. Among his observations on the case of Master Todd, related by Dr. Rush in your last Number, is the following passage. "The having been bitten by a dog, affords, as I have before observed, of itself, no presumption of the nature of the disease, unless the dog was proved to be mad. That this was the case in the instance before us, *there is no attempt to show.*" What will your numerous and respectable readers say to all this, when they find that Dr. Rush has expressly stated the most unequivocal confirmation of the madness of the dog in these words. The same dog which bit Mr. Todd's son, bit at the same time, a cow, a pig, a dog, and a black servant of Mr. Todd's. The cow and pig died; the dog *became mad*, and was killed by his master." If this be not proof sufficient to convince the most sturdy sceptic, where are we to look for better?

A critic should stand on commanding ground, such as to enable him to have a comprehensive view of his subject in all its bearings; it is therefore greatly to be desired, as you have solicited the future correspondence of C. B. that the next time he takes the trouble to write for the information and edification of his professional brethren, he will remember the advice of Horace.

(Scribendi recte, sapere est principium et fons) and deem it a primary qualification to understand the subject himself. I am, &c.

CHARLES ARMSTRONG.

*Upper Charlotte Street, Fitzroy Square,*
*November* 13, 1808.

